# Supplementary material for: A set of multi-entry identification keys to African frugivorous flies (Diptera, Tephritidae)
Source: Zookeys. 2014 Jul 24;(428):97–108. doi: 10.3897/zookeys.428.7366 (PMC4143993; doi:10.3897/zookeys.428.7366)
Supplement: Supplementary material 4 — Key to Capparimyia [file zookeys-428-097-s004.zip › SF4_ZooKeys_key to Capparimyia/lucid_player/help/help/what_it_does.htm]

What does the Lucid3 Player do? 


# What does the Lucid3 Player do?

The Lucid3 Player is an effective and powerful identification and diagnostic
tool. It is used to play interactive identification or diagnostic keys
or guides created using the Lucid3 Builder1.

Lucid3 keys are usually created by an expert for a particular group
of organisms or things (such as fruit flies, frogs, wildflowers, corals,
grasses, crop diseases, mouth ulcers or minerals). The key comprises a
list of all the entities (e.g. species or other taxa, objects, diseases
etc) in the group, a list of the features that may be used to identify
them, and a matrix of scores of the entities for the features.

When a key is deployed in the Lucid3 Player, it can be used to identify
specimens that belong to the group covered. Specimens are described by
selecting, from the list of all features provided in the key, those features
that match the specimen. At each step, the Lucid Player sorts the list
of entities in the key into those that match the developing description
and those that do not. As features are progressively chosen, the list
of matching entities will be progressively reduced until a single entity
(or perhaps a short list of entities) remains. The identification is complete.

Identifying the specimen – finding a name for it – is only the first
step in finding out more about it. Links from the key’s entities provide
access to fact sheets and other information provided by the key builder.
A name is also the starting point for finding information on an organism
beyond the key, using the World Wide Web and other resources.

The Lucid3 Player provides other tools and information to help with
the identification. For example, features in Lucid keys are usually provided
with explanatory notes and images to help learn about them. Sub-sets
of features can help organise the list of features to make it easier to
manage, and the Player’s Best function is a powerful
tool to help decide which feature to address next.

View the topic Using the Lucid3 Player
next, for an introduction to the look and feel of the Player.

1 If you think you may like
to build your own Lucid3 key, see the topic About
the Lucid3 Builder Or visit www.lucidcentral.org,
for more information.
